# Resveratrol Enhances Autophagic Flux and Promotes Ox-LDL Degradation in HUVECs via Upregulation of SIRT1

**DOI:** 10.1155/2016/7589813

**Published:** 2016-03-16

**Authors:** Yanlin Zhang, Xueqin Cao, Wawa Zhu, Zhihua Liu, Huihui Liu, Yande Zhou, Yongjun Cao, Chunfeng Liu, Ying Xie

**Affiliations:** ^1^Department of Neurology, The Second Affiliated Hospital of Soochow University, Suzhou 215004, China; ^2^Department of Endocrinology, The Second Affiliated Hospital of Soochow University, Suzhou 215004, China; ^3^Department of Endocrinology, Ningbo Number 2 Hospital, Ningbo 315000, China; ^4^Institute of Neuroscience, Soochow University, Suzhou 215123, China

## Abstract

Oxidized low-density lipoprotein- (Ox-LDL-) induced autophagy dysfunction in human vascular endothelial cells contributes to the development of atherosclerosis (AS). Resveratrol (RSV) protects against Ox-LDL-induced endothelium injury. The objective of this study was to determine the mechanisms underlying Ox-LDL-induced autophagy dysfunction and RSV-mediated protection in human umbilical vein endothelial cells (HUVECs). The results showed that Ox-LDL suppressed the expression of sirtuin 1 (SIRT1) and increased LC3-II and sequestosome 1 (p62) protein levels without altering p62 mRNA levels in HUVECs. Pretreatment with bafilomycin A1 (BafA1) to inhibit lysosomal degradation abrogated the Ox-LDL-induced increase in LC3-II protein level. Ox-LDL increased colocalization of GFP and RFP puncta in mRFP-GFP-tandem fluorescent LC3- (tf-LC3-) transfected cells. Moreover, Ox-LDL decreased the expression of mature cathepsin D and attenuated cathepsin D activity. Pretreatment with RSV increased the expression of SIRT1 and LC3-II and increased p62 protein degradation. RSV induced RFP-LC3 aggregation more than GFP-LC3 aggregation. RSV restored lysosomal function and promoted Ox-LDL degradation in HUVECs. All the protective effects of RSV were blocked after SIRT1 was knocked down. These findings demonstrated that RSV upregulated the expression of SIRT1, restored lysosomal function, enhanced Ox-LDL-induced impaired autophagic flux, and promoted Ox-LDL degradation through the autophagy-lysosome degradation pathway in HUVECs.

## 1. Introduction

Autophagy is a dynamic process where aberrant intracellular organelles and macromolecules, including accumulated lipids, are sequestered into double-membrane vesicles called autophagosomes to be delivered to and fused with lysosomes, wherein they are degraded by lysosomal enzymes, and the eventual recycling of macromolecules occurs [1, 2]. Lysosomal degradation is involved in the terminal steps of autophagy. This function is dependent on lysosomal proteases, most importantly, the cysteine cathepsins and the aspartic protease cathepsin D [3]. The term* autophagic flux* encompasses the entire process of autophagy including the delivery of cargo to lysosomes, its subsequent breakdown, and release of the resulting macromolecules back into the cytosol [4]. Cathepsin D deficiency results in dysfunction of lysosomal processes, which in turn impairs autophagic flux [5]. Moreover, the quantification of autophagosomes to assess autophagic flux can be misleading, since an increase in the amount of LC3-II or accumulation of autophagosomes may reflect induction of autophagy, reduction in autophagosome turnover, or the inability of turnover to keep pace with increased autophagosome formation [6].

Oxidized low-density lipoprotein (Ox-LDL) causes vascular endothelial dysfunction by triggering lipid accumulation, local inflammation, and toxic events and has a key role in the pathogenesis of atherosclerosis (AS) and atherosclerotic plaque rupture [7]. Several lines of evidence suggest that autophagy dysfunction by oxidized lipids is observed in advanced atherosclerotic plaques [8]. Our previous study showed that the autophagy-lysosome pathway in HUVECs is activated by Ox-LDL [9]. Whether the autophagic flux process is completed after treatment of HUVECs with Ox-LDL is unknown.

SIRT1, a member of the NAD+-dependent deacetylases [10], plays an important role in enhancing autophagic flux in cardiomyocytes [11]. It has also been reported that the SIRT1 activator resveratrol (RSV) improves cardiac dysfunction in diabetic mice by increasing autophagic flux, dependent on activated SIRT1 [12]. RSV is a phenolic natural component of* Vitis vinifera* L., mainly found in the skin of grapes and red wine. Evidence indicates that this natural compound displays many pharmacologic effects, including a modulatory effect on lipoproteins, anti-inflammatory and antioxidative effects, and attenuation of endothelial dysfunction, an initial step in AS progression [13, 14]. SIRT1 regulation of autophagy is essential for the cytoprotective effects of RSV [15].

This study was performed to observe the effect of Ox-LDL on autophagic flux of HUVECs and investigate whether RSV could enhance the injured autophagic flux and reduce Ox-LDL accumulation in HUVECs, as well as the role of SIRT1 in this RSV effect.

## 2. Materials and Methods

### 2.1. Reagents and Antibodies

Ox-LDL, Dil-Ox-LDL, and Cell Counting Kit-8 (CCK-8) were purchased from Yiyuan Biotechnologies (Guangzhou, China). RSV (R5010), BafA1 (B1793), Dulbecco's Modified Eagle's Medium (DMEM), and primary antibodies against *β*-actin, p62, *β*-tubulin, and other chemicals were purchased from Sigma-Aldrich (St. Louis, MO, USA). Primary antibodies against SIRT1 were obtained from Santa Cruz (Dallas, TX, USA) and those against cathepsin D, LC3, and LAMP1 and the cathepsin D activity assay kit (ab65302) were purchased from Abcam (Cambridge, UK). Fetal bovine serum (FBS) was purchased from Hyclone (GE Healthcare, Buckinghamshire, UK).

### 2.2. Cell Culture

HUVECs were purchased from Shanghai Gene Chemical Co., Ltd. (Shanghai, China). Cells were cultured in culture plates with high-glucose DMEM supplemented with 10% FBS and 1% penicillin and streptomycin at 37°C in a humidified atmosphere containing 5% CO_2_/95% air. All experiments were performed following 3–10 passages when the cells reached ~80–90% confluence. To avoid the induction of autophagy through serum starvation, all experiments were performed in complete culture medium.

### 2.3. Cell Viability Measurement

CCK-8 was used to measure cell viability according to the manufacturer's instructions. Briefly, HUVECs were seeded in a 96-well microplate at an appropriate density of cells/well and then pretreated with Ox-LDL at a series of concentrations (0, 30, 60, 90, and 120 *μ*g/mL) or pretreated with RSV at a series of concentrations (0, 12.5, 25, 50, and 100 *μ*M). Subsequently, CCK-8 solution (10 *μ*L/well) was added to the wells, the plate was incubated at 37°C for 1.5 h, and the absorbance was determined with a microplate reader (Tecan, Mannedorf, Switzerland) at a wavelength of 490 nm. The optical density value was reported as the percentage of cell viability in relation to the control group.

### 2.4. Flow Cytometry Analysis of Dil-Ox-LDL in HUVECs

Cultures of HUVECs were incubated with 60 *μ*g/mL Dil-Ox-LDL for 12 h at 37°C. The cells were gently washed three times with 1x phosphate-buffered saline (PBS) and then digested with trypsinization for 1 min. Finally, the cells were resuspended in a volume of 300 *μ*L of 1x PBS. Dil-Ox-LDL in HUVECs was detected by a flow cytometer (FC500, Beckman Coulter, Miami, FL, USA) at excitation-emission wavelengths of 549 nm and 565 nm.

### 2.5. Western Blot Analysis

Cell lysates were prepared using lysis buffer (150 mM NaCl, 25 mM Tris, 5 mM EDTA, and 1% Nonidet P-40, pH 7.5) with protease inhibitor cocktail tablets (Roche Diagnostics, Penzberg, Germany). Ten percent sodium dodecyl sulfate-polyacrylamide gels were used to separate the protein samples and then transferred onto polyvinylidene fluoride membranes (Millipore, Bedford, MA, USA). Subsequently, membranes were blocked with 5% (w/v) skimmed dry milk powder dissolved by Tris-buffered saline containing 0.1% (v/v) Tween 20 (TBST) for 1 h and incubated with primary antibodies at 4°C for 24 h. After that, membranes were washed with TBST every ten minutes three times and then incubated with secondary antibodies for 1 h. Specific proteins were detected using a chemiluminescence kit (GE Healthcare, Buckinghamshire, UK). The densitometric analysis was performed using Image J software (National Institute of Health, Bethesda, MD, USA).

### 2.6. Reverse Transcription PCR (RT-PCR)

Total RNA was extracted with Trizol (Invitrogen, Carlsbad, CA, USA) according to the manufacturer's instructions. Total RNA (1 g) of each sample was reverse transcribed into cDNA by using a cDNA synthesis kit (Fermentas, Vilnius, Lithuania). The resultant cDNA was amplified with gene-specific primers (Genscript, Nanjing, China) using the PCR Master Mix kit (Fermentas, Vilnius, Lithuania). The human-specific PCR primers were for p62 (forward: CTGCCCAGACTACGACTTGTGT; reverse: TCAACTTCAATGCCCAGAGG) and for GAPDH (forward: GUAUGACAACAGCCUCAAGTT; reverse: 5′-CUUGAGGCUGUUGUCAUACTT). PCR products were separated on 2% agarose gels. The optical band densities were analyzed with Image J software (National Institute of Health, Bethesda, MD, USA).

### 2.7. Confocal Microscopy

HUVECs were transfected with 0.8 *μ*g plasmids expressing tandem fluorescent RFP-GFPLC3 (Add gene, Cambridge, MA, USA) for 24 h, and then cells were treated with Ox-LDL or RSV for another 12 h. Next, cells were fixed with 4% (w/v) paraformaldehyde for 8 min and coverslips were mounted with mounting medium using DAPI (Vector Laboratories, Burlingame, CA, USA). Images were taken and processed on a confocal microscope system (LSM 700, Carl Zeiss Inc., Oberkochen, Germany). Acquisition software used was Zen 2011, Expert mode (Carl Zeiss Inc., Germany).

### 2.8. Cathepsin D Activity Assay

The cathepsin D assay was performed using a fluorometric cathepsin D activity assay kit from Abcam (ab65302) according to the manufacturer's instruction. Briefly, cells were harvested and then dissolved with the cell lysis buffer provided in the kit. Protein concentration was determined by the BCA method; 50 ng of protein was incubated with the cathepsin D substrate mixture at 37°C for 1 h. Fluorescence intensity of the synthetic substrate treated with cathepsin D was measured using a fluorescence plate reader (Infinite M200 PRO, Tecan) at Ex/Em = 328/460 nm.

### 2.9. Transient Transfection

siRNAs for SIRT1 (human) (5′-CGGGAAUCCAAAGGAUAAUTT-3′ and 5′-AUUAUCCUUUGGAUUCCCGTT-3′) were purchased from GenePharma (Shanghai, China). The siRNA duplexes were transfected using Lipofectamine 3000 reagent (Life Technologies, Carlsbad, CA, USA). Transfection efficiency was determined by western blotting 24 h after transfection.

### 2.10. Statistical Analysis

Three different HUVEC batches were studied. All date are expressed as mean ± standard error of the mean. Statistical analysis was performed by SPSS v17.0 (SPSS, Chicago, IL, USA) and GraphPad Prism v5.0 using Student's *t*-test or one-way analysis of variance followed by Tukey* post hoc* analysis where applicable. A *p* value <0.05 was considered statistically significant.

## 3. Results

### 3.1. Ox-LDL Decreased SIRT1 Expression and Impaired Autophagic Flux in a Dose- and Time-Dependent Manner in HUVECs

**Figure 1:**
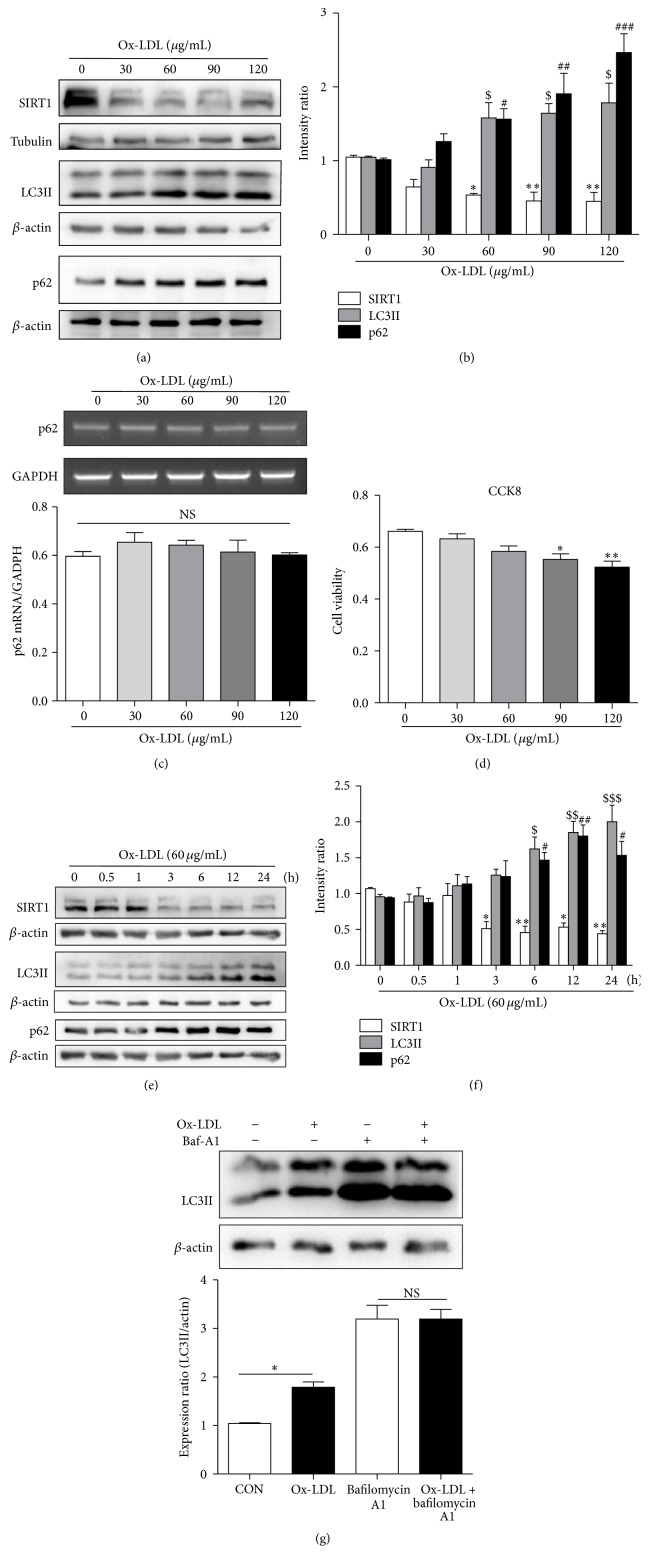
Ox-LDL suppressed SIRT1 expression and impaired autophagic flux in HUVECs. Western blotting analysis showed that Ox-LDL decreased SIRT1, increased LC3-II, and increased p62 in a dose-dependent manner after Ox-LDL treatment of HUVECs for 12 h (a, b). Actin served as loading controls. (c) The relative mRNA level of p62 assessed by reverse transcription PCR. (d) Cell viability was determined by a CCK-8 assay; cell survival was given as percentage of control. Western blotting analysis showed that Ox-LDL (60 *μ*g/mL) decreased SIRT1, increased LC3-II, and increased p62 in a time-dependent manner in HUVECs (e, f). (g) HUVECs were treated with 60 *μ*g/mL Ox-LDL in the presence of Baf A1 (50 nM), and LC3-II level was evaluated by western blot analysis. Data are expressed as mean ± SEM; *n* = (3–6); ^*∗*^ *p* < 0.05, ^*∗∗*^ *p* < 0.01 versus controls of SIRT1; ^$^ *p* < 0.05, ^$$^ *p* < 0.01, and ^$$$^ *p* < 0.001 versus controls of LC3II; ^#^ *p* < 0.05, ^##^ *p* < 0.01, and ^###^ *p* < 0.001 versus controls of p62; NS: not significant.

Ox-LDL treatment decreased the protein levels of SIRT1, while it increased those of LC3-II and the autophagic substrate p62 in a dose-dependent manner in HUVECs (Figures 1(a) and 1(b)). The mRNA level of p62 did not increase after Ox-LDL treatment (Figure 1(c)). Since the results of the CCK8 assay indicated that cell viability significantly decreased in response to high concentrations of Ox-LDL (90, 120 *μ*g/mL; Figure 1(d)), cell death resulted in the inactivation of the autophagic programme which inhibited autophagy [16], so 60 *μ*g/mL Ox-LDL was selected as the optimal concentration for the subsequent experiments to explore the effect of Ox-LDL on autophagic flux rather than cell death induced autophagy inhibition in HUVECs. Ox-LDL decreased the expression of SIRT1 and upregulated that of LC3-II and p62 in a time-dependent manner (Figures 1(e) and 1(f)). We therefore chose 12 h as the time point to assess Ox-LDL's effects in the following experiments. To explore whether the Ox-LDL-induced increase in the amount of LC3-II was owing to increased autophagy initiation or decreased degradation, we pretreated cells with BafA1 (50 nM) for 30 min to inhibit autophagic flux and subsequently incubated the HUVECs with 60 *μ*g/mL Ox-LDL for 12 h; the Ox-LDL-induced increase in LC3-II was nullified in the presence of BafA1 (Figure 1(g)). These results demonstrated that Ox-LDL suppressed SIRT1 expression and impaired autophagic flux in HUVECs.

### 3.2. RSV Activated SIRT1 and Enhanced Autophagic Flux in a Dose- and Time-Dependent Manner in HUVECs

**Figure 2:**
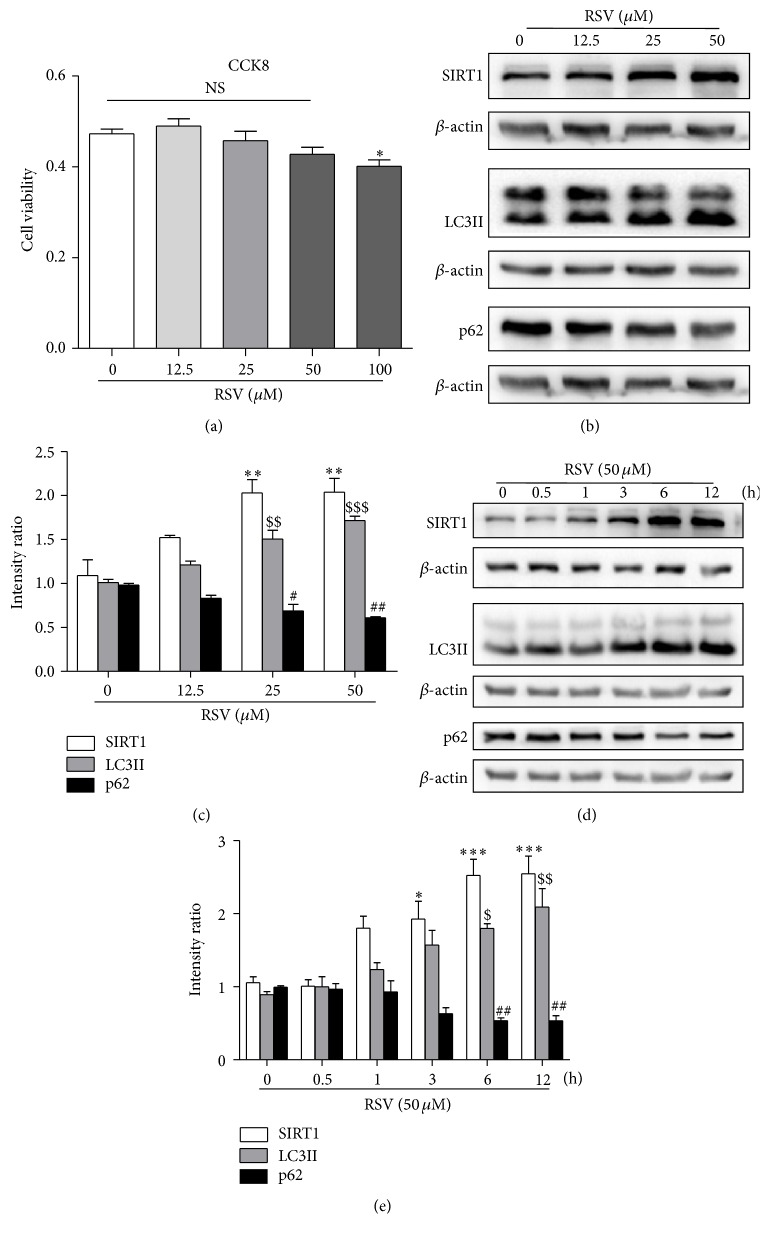
Resveratrol increased the expression of SIRT1 and enhanced autophagic flux in HUVECs. (a) Cell viability was determined by CCK-8 assay after treatment of HUVECs with different concentrations of resveratrol for 12 h. Western blotting analysis showed that resveratrol increased the expression of SIRT1 and LC3-II and decreased that of p62 in a dose-dependent manner (b, c). Resveratrol (50 *μ*M) increased the expression of SIRT1 and LC3-II and decreased that of p62 in a time-dependent manner (d, e). Actin served as loading controls. Data are expressed as mean ± SEM; *n* = (3–7); ^*∗*^ *p* < 0.05, ^*∗∗*^ *p* < 0.01, and ^*∗∗∗*^ *p* < 0.001 versus controls of SIRT1; ^$^ *p* < 0.05, ^$$^ *p* < 0.01, and ^$$$^ *p* < 0.001 versus controls of LC3II; ^#^ *p* < 0.05, ^##^ *p* < 0.01 versus controls of p62; NS: not significant.

To determine whether RSV could induce autophagy in HUVECs with a single treatment, we investigated the levels of LC3-II and p62. Low concentrations of RSV (0, 12.5, 25, and 50 *μ*M) did not harm the cell viability of HUVECs, while treatment with 100 *μ*M significantly decreased cell viability (Figure 2(a)) compared with the control. Next, we analyzed the effect of low concentrations of RSV on autophagic flux. RSV increased the protein levels of SIRT1 and LC3-II and decreased p62 in a dose-dependent manner in HUVECs (Figures 2(b) and 2(c)). We therefore selected 50 *μ*M RSV as the experimental concentration to be used in the following experiments. 50 *μ*M RSV increased the protein levels of SIRT1 and LC3-II and decreased that of p62 in a time-dependent manner (Figures 2(d) and 2(e)). These results demonstrated that a single treatment with RSV enhanced autophagic flux in HUVECs.

### 3.3. RSV Ameliorated Ox-LDL-Induced Impairment of Autophagic Flux and Reduced the Accumulation of Dil-Ox-LDL in HUVECs

**Figure 3:**
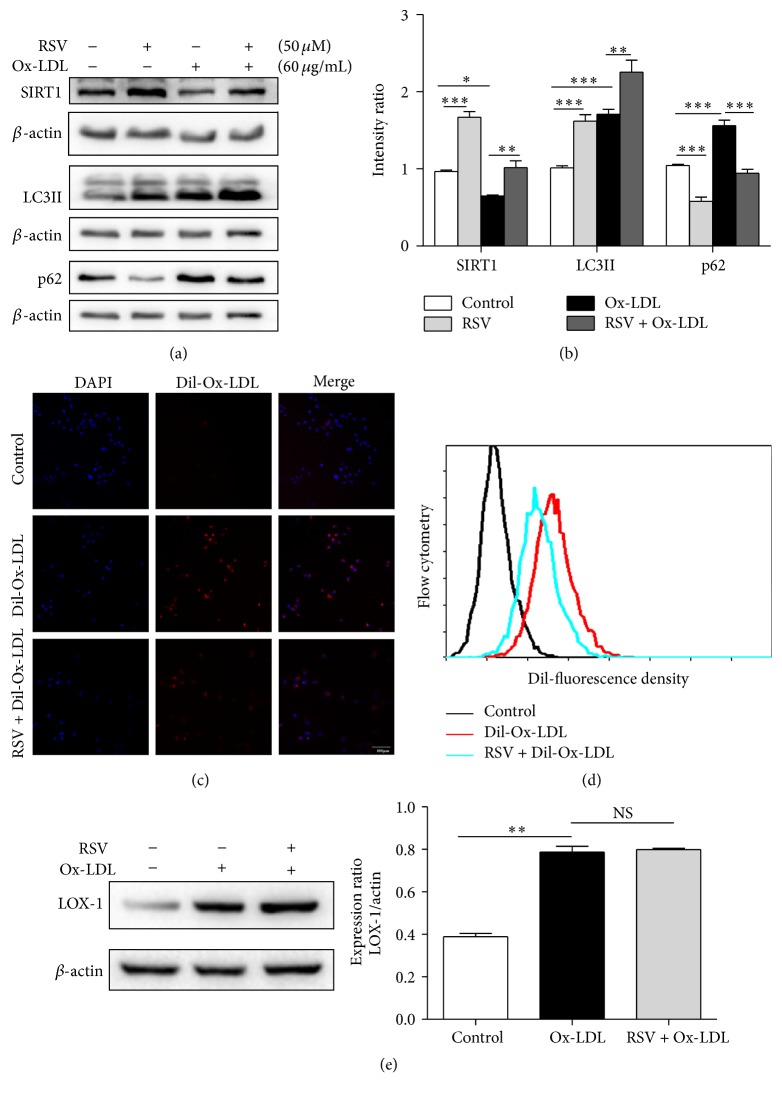
Resveratrol ameliorates Ox-LDL-induced impaired autophagic flux and reduces the accumulation of Ox-LDL in HUVECs. Western blotting analysis of SIRT1, LC3-II, and p62 after HUVEC pretreatment with 50 *μ*M resveratrol for 1 h and subsequently incubation with Ox-LDL (60 *μ*g/mL) for 12 h (a, b). Data are expressed as mean ± SEM; *n* = 3; ^*∗*^ *p* < 0.05, ^*∗∗*^ *p* < 0.01, and ^*∗∗∗*^ *p* < 0.001 versus controls; NS: not significant. (c) The accumulation of Dil-Ox-LDL in HUVECs was determined by confocal microscopy. Scale bar: 100 *μ*m. (d) Flow cytometry detected the fluorescence density of Dil-Ox-LDL in HUVECs. (e) Western blotting analysis of LOX-1 after cells was treated with Ox-LDL or RSV; data are expressed as mean ± SEM; *n* = 3; ^*∗∗*^ *p* < 0.01 versus control; NS: not significant.

To investigate whether RSV could ameliorate the Ox-LDL-induced impairment in autophagic flux, we pretreated HUVECs with RSV (50 *μ*M) for 1 h and subsequently incubated them in Ox-LDL (60 *μ*g/mL) for 12 h. The protein levels of SIRT1 and LC3-II were increased, and p62 was decreased compared with Ox-LDL treatment alone (Figures 3(a) and 3(b)). At the same time, the results of confocal microscopy and flow cytometry indicated that pretreatment with 50 *μ*M RSV for 1 h significantly decreased the accumulation of intracellular Dil-Ox-LDL (Figures 3(c) and 3(d)). Next, we explored whether the decreased accumulation of Ox-LDL was due to RSV reducing the uptake of Ox-LDL. Lectin-like oxidized low-density lipoprotein-1 (LOX-1) is the major receptor for Ox-LDL uptake in endothelial cells. The results of western blotting showed that treatment with Ox-LDL increased the expression of LOX-1. However, pretreatment with 50 *μ*M RSV did not decrease the amount of LOX-1 compared with Ox-LDL treatment alone (Figure 3(e)). These results suggested that treatment with RSV ameliorates Ox-LDL-induced impaired autophagic flux and reduces the accumulation of Dil-Ox-LDL in HUVECs.

### 3.4. Lysosomal Dysfunction Contributes to Ox-LDL-Induced Disruption of Autophagic Flux in HUVECs

**Figure 4:**
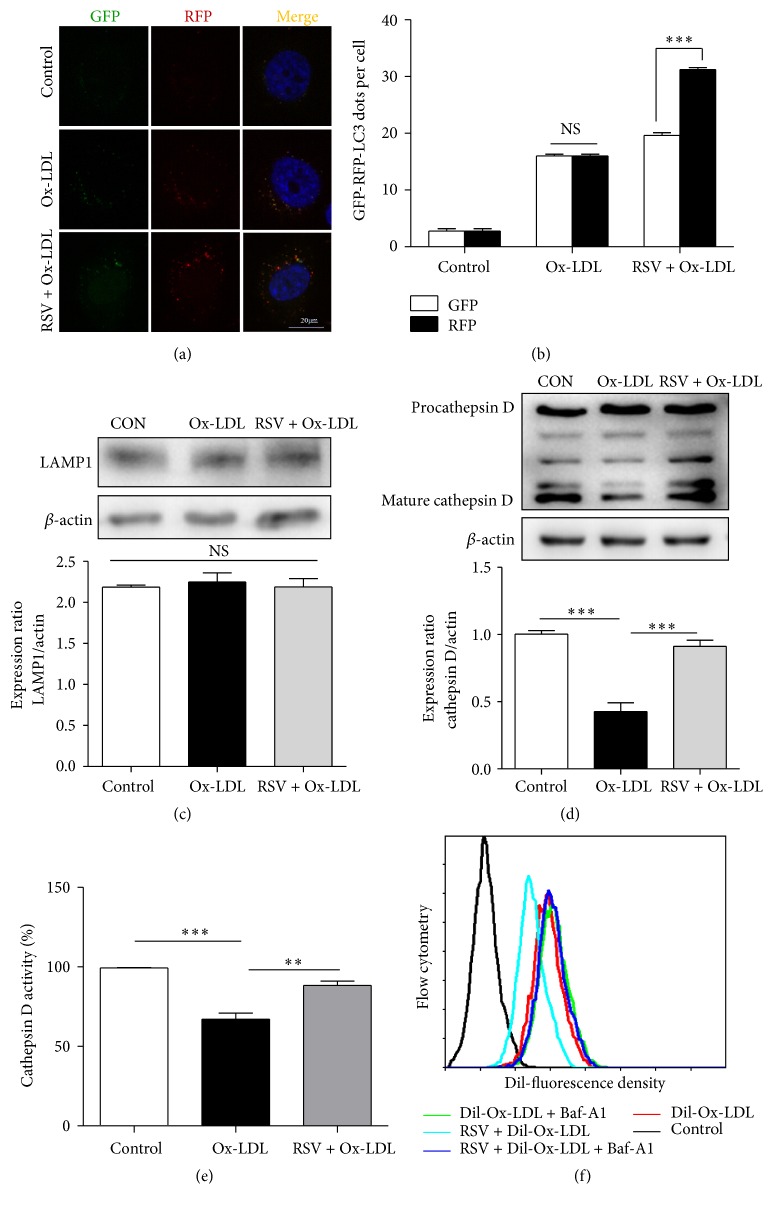
Resveratrol restored lysosomal dysfunction caused by Ox-LDL in HUVECs. (a) HUVECs were transfected with RFP-GFP-tandem fluorescent LC3 cDNA for 24 h, followed by resveratrol treatment for 1 h, and subsequently incubated with Ox-LDL for another 12 h. (b) LC3 dots were determined by fluorescent confocal microscopy and quantified. At least 30 cells per group were included for the counting of RFP- and GFP-LC3 puncta/cell. Scale bar: 20 *μ*m; ^*∗∗∗*^ *p* < 0.001 versus GFP; NS: not significant. Western blotting analysis of the protein level of LAMP1 (c) and cathepsin D (d) after HUVEC pretreatment with 50 *μ*M resveratrol for 1 h, and subsequently incubation with Ox-LDL (60 *μ*g/mL) for 12 h. Data are expressed as mean ± SEM; *n* = 3; ^*∗∗∗*^ *p* < 0.001 versus corresponding controls; NS: not significant. (e) Cathepsin D activity in HUVECs was evaluated by a fluorometric cathepsin D activity assay kit. Data are expressed as mean ± SEM; *n* = 3; ^*∗∗*^ *p* < 0.01; ^*∗∗∗*^ *p* < 0.001. (f) Flow cytometry detected the fluorescence density of Dil-Ox-LDL after HUVECs were treated with Dil-Ox-LDL and RSV in the presence or absence of Baf A1.

Tf-LC3 was used to investigate the autolysosome maturation process. mRFP is more stable than GFP in the acidic/proteolytic environment of the lysosome. Therefore, the normal maturation of autolysosomes is characterized with increased red-only puncta, while in contrast, colocalization of GFP and RFP puncta would indicate disruption of autophagic flux [17]. Here we utilized this system and established the pattern of GFP and mRFP fluorescence changes in HUVECs after Ox-LDL and RSV treatment. Ox-LDL induced the colocalization of GFP and RFP puncta; this result further indicates that Ox-LDL impaired autophagic flux. However, pretreatment with 50 *μ*M RSV for 1 h followed by incubation of the HUVECs in 60 *μ*g/mL Ox-LDL for 12 h led to a significant generation of GFP and mRFP puncta, and the GFP puncta were much reduced compared with mRFP puncta, indicating efficient autophagic flux (Figures 4(a) and 4(b)). Next, we explored whether Ox-LDL inhibition of autophagic flux was due to lysosomal dysfunction. Our results showed that the expression of a lysosomal membrane protein (LAMP1) was not altered after treatment with Ox-LDL and RSV (Figure 4(c)). This indicated that Ox-LDL does not reduce the number of lysosomes. Then, we examined the protein levels of cathepsin D. Cathepsin D is an aspartic lysosomal enzyme, which plays an essential role in the degradation process of autophagy. The results of western blotting indicated that the active form of cathepsin D (mature cathepsin D) was significantly decreased after treatment with Ox-LDL and that RSV restored the expression of cathepsin D (Figure 4(d)). Consistent with the western blot data, Ox-LDL significantly lowered cathepsin D enzyme activity, while RSV was able to restore cathepsin D enzyme activity (Figure 4(e)). To investigate whether RSV decreased the accumulation of intracellular Ox-LDL through lysosomal degradation, we pretreated the cells with BafA1 to inhibit the fusion of autophagosomes and lysosomes; the protective effect of RSV in terms of decreasing the accumulation of Ox-LDL was blocked (Figure 4(f)). All these results demonstrated that Ox-LDL-induced lysosomal dysfunction contributes to the impaired autophagic flux. RSV restored the function of lysosomes, resulting in complete autophagic flux, and therefore decreased the accumulation of intracellular Ox-LDL through autophagy-lysosome pathway-mediated degradation.

### 3.5. SIRT1 Is Essential for RSV's Amelioration of Ox-LDL-Induced Impaired Autophagic Flux and Promotes the Degradation of Ox-LDL in HUVECs

**Figure 5:**
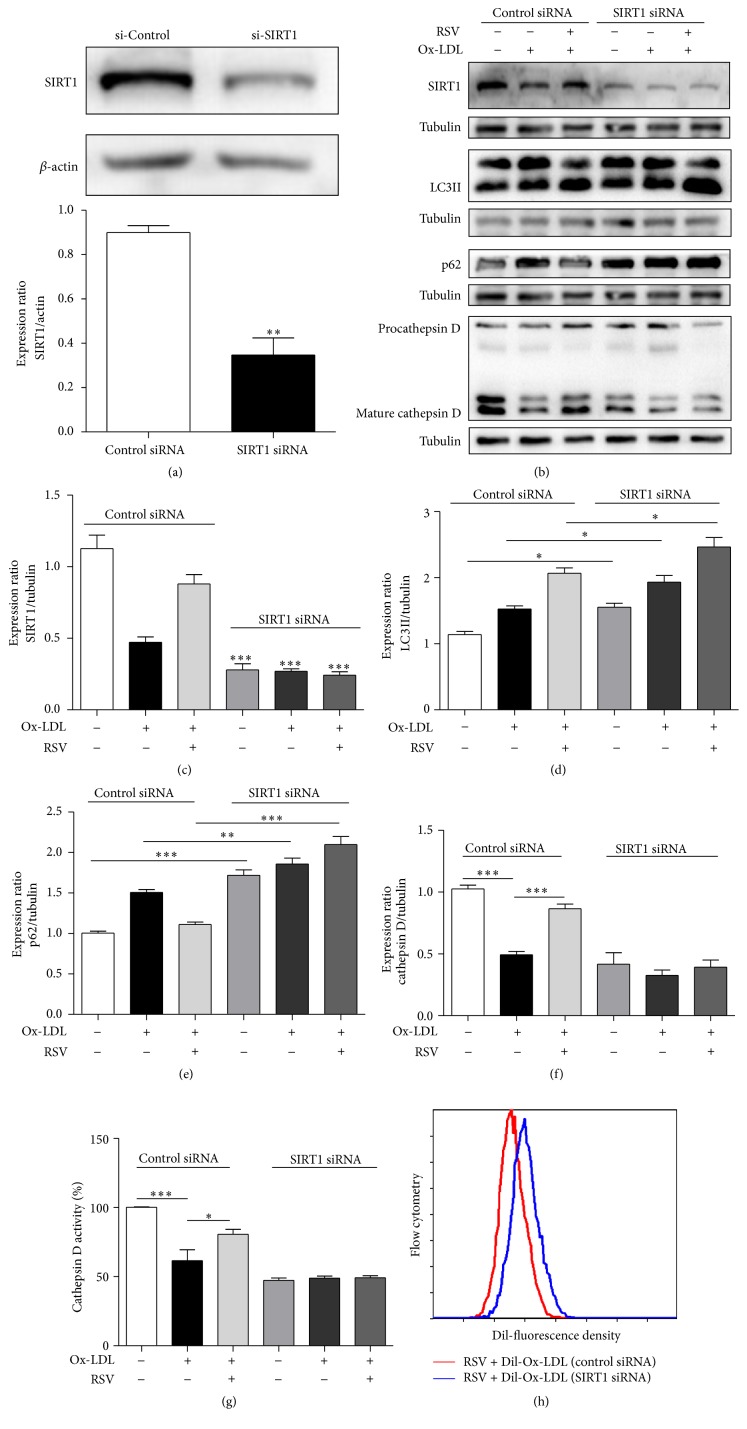
Resveratrol amelioration of autophagic flux in HUVECs in the presence of Ox-LDL is dependent on SIRT1. (a) Western blot analysis of SIRT1 in HUVECs transfected with SIRT1 or nontargeting siRNA. Pretreatment with resveratrol (50 *μ*M) subsequently incubated with Ox-LDL (60 *μ*g/mL) for 12 h after SIRT siRNA transfection. Western blot analysis of SIRT1, LC3-II, p62, and cathepsin D (b–f). Cathepsin D activity was detected with a cathepsin D activity assay kit (g). Data are expressed as mean ± SEM; *n* = 5; ^*∗*^ *p* < 0.05, ^*∗∗*^ *p* < 0.01, and ^*∗∗∗*^ *p* < 0.001 versus corresponding controls. Cells were transfected with SIRT1 or nontargeting siRNA for 24 h and then treated with Dil-Ox-LDL for 12 h; flow cytometry detected the fluorescence density of Dil-Ox-LDL in HUVECs (h).

To investigate whether RSV's ability to ameliorate impaired autophagic flux and promote the degradation of Ox-LDL depends on SIRT1, we used siRNA to knock down SIRT1. The protein level of SIRT1 significantly decreased after transfection with SIRT1 siRNA (Figures 5(a), 5(b), and 5(c)). After transfection with SIRT1 siRNA, the protective effects of RSV on the expression of LC3-II and p62 were abolished (Figures 5(b), 5(d), and 5(e)). Similarly, the protective effects of RSV on the expression and activity of mature cathepsin D were also abolished by SIRT1 siRNA (Figures 5(b), 5(f), and 5(g)). The results of flow cytometry showed that RSV could not reduce the accumulation of Dil-Ox-LDL after SIRT1 knockdown (Figure 5(h)). These results indicated that SIRT1 is essential for RSV's amelioration of Ox-LDL-induced impaired autophagic flux and the degradation of Ox-LDL in HUVECs.

## 4. Discussion 

Ox-LDL-induced vascular endothelial dysfunction is a driving force in the initiation and development of AS [18–20]. RSV has emerged as a drug candidate for the prevention and treatment of vascular diseases because of its ability to improve endothelial function [2, 21, 22]. Atherosclerotic regions are characterized by accumulation of large amounts of Ox-LDL in macrophages, vascular smooth muscle cells, and endothelial cells [23]. Enhanced autophagy attenuates Ox-LDL-induced lipid aggregation in macrophages and smooth muscle cells [24, 25].

The autophagy-lysosomal system is crucial in the processing and clearance of intracellular lipids [2], and dysregulation of autophagy might contribute to the development of atherosclerosis [26]. Our previous study had demonstrated that Ox-LDL activated the autophagy-lysosome pathway in HUVECs [9]; however, the conversion of LC3-I to LC3-II does not necessarily result in complete autophagy since LC3II could be degraded by p62 in the later stages [27]. Several studies have reported on the role of Ox-LDL in autophagy, as Ox-LDL was reported to increase the amount of LC3-II and p62 in HUVECs and macrophages, and found that increased p62 may be due to Ox-LDL inhibition of autophagic flux [13, 28–30]. It has also been reported that excessive lipid concentrations may inhibit autophagic flux by impairing lysosomal acidification and hydrolase activity in *β*-cells [31]. However, whether the autophagic flux process is completed after treatment of HUVECs with Ox-LDL and the underline mechanism is still unknown. In our present study, we analyzed the changes in the amounts of LC3-II and p62. Our results showed that Ox-LDL increased the protein levels of LC3-II and p62, but without increasing the mRNA levels of p62. P62 serves as the autophagic substrate; therefore, the increase in p62 protein levels in the presence of Ox-LDL may be due to impaired autophagic degradation. Conversion of LC3-I to LC3-II is essential for the formation of autophagosomes and can serve as a marker of autophagy. However, our results showed that, compared with treatment with bafilomycin only, Ox-LDL did not increase the amount of LC3-II when administered with BafA1. Bafilomycin was able to block the fusion of autophagosomes with lysosomes, and the results indicated that the initiation of autophagosomes was not increased. Moreover, in Tf-LC3-transfected cells, Ox-LDL significantly increased colocalization of GFP and RFP puncta, which may be due to impaired autophagic flux. All these results suggested that Ox-LDL had not increased the initiation of autophagy but rather impaired autophagic flux in HUVECs.

Lysosomes have the ability to process both exogenous material, including lipids, and autophagy-derived cargo such as dysfunctional proteins/organelles. The most important proteinase for LDL degradation is cathepsin D [32]. Ox-LDL inhibited the breakdown of the added Ox-LDL and other lipoproteins at the level of lysosomal degradation by direct inactivation of the lysosomal protease in macrophages [33]. Ox-LDL also blocked the maturation of cathepsin D in retinal pigment epithelium [34]. Emanuel et al. demonstrated that Ox-LDL markedly impaired lysosomal function in macrophages, causing aberrant lysosomal engorgement, increased lysosomal pH, and decreased proteolytic capacity; lysosomal dysfunction may contribute to the inhibition of autophagic flux [30]. In our present study, we found that Ox-LDL decreased the enzymatic activity of cathepsin D and the protein levels of mature cathepsin D in HUVECs, without altering the expression of LAMP1. These results suggested that Ox-LDL does not change the number of lysosomes but rather attenuates lysosomal biological function in HUVECs, which contributes to the impaired autophagic flux and ultimately leads to the accumulation of Ox-LDL.

The SIRT1 activator RSV improves cardiac dysfunction in diabetic mice in a process associated with enhancing autophagic flux through the SIRT1/FOXO1/Rab7 axis [12]. In the present study, our results showed that RSV increased the protein levels of LC3-II and decreased p62 and increased intracellular puncta of RFP-LC3, which suggested the presence of completed autophagic flux. RSV restored the function of lysosomes through an increase of the protein expression and activity of mature cathepsin D, which contributes to the completed autophagic flux. Meanwhile, RSV decreased the accumulation of Ox-LDL in HUVECs. LOX-1 is the major receptor for Ox-LDL uptake in endothelial cells [35]. The expression of LOX-1 was found to be upregulated in the presence of Ox-LDL [36], and similar results were observed in our present study. Ox-LDL increased the amount of LOX-1; however, RSV did not decrease its expression; therefore, the reduced accumulation of Ox-LDL was due to RSV-induced degradation of Ox-LDL rather than reduced uptake of Ox-LDL. Moreover, when cells were pretreated with bafilomycin A1 to inhibit the autophagic flux, the protective effects of RSV in terms of reducing the accumulation of intracellular Ox-LDL were blocked. Therefore, the protective effects of RSV are associated with amelioration of Ox-LDL-induced impaired autophagic flux, and RSV-induced restoration of lysosomal function contributes to the completed autophagic flux in HUVECs.

Autophagy regulated by SIRT1 protects HUVECs against Ox-LDL-induced injury [37]. SIRT1-mediated autophagy also attenuates lipid accumulation in hepatocytes [38], and SIRT1 was demonstrated to increase autophagic flux through deacetylation of essential autophagic modulators such as ATG5 and ATG7 [39]. SIRT1-mediated deacetylation of FoxO1 also plays a key role in enhancing autophagic flux in cardiomyocytes [11]. RSV, a pharmacological SIRT1 activator, protects HUVECs from Ox-LDL-induced injury by upregulating the AMPK/SIRT1 or CAMP-PRKA-AMPK-SIRT1 signaling pathways [13–15]; whether RSV protects HUVECs through enhancing autophagic flux is not clear. It is reported that RSV improves cardiac dysfunction in diabetic mice associated with enhanced autophagic flux through the SIRT1/FOXO1/Rab7 axis [12]. Therefore, SIRT1 plays an important role in the regulation of autophagy and autophagic flux. The purpose of this study was to explore whether RSV protects HUVECs from Ox-LDL-induced injury through enhancing autophagic flux dependent on SIRT1 and further investigate the mechanism of RSV protective effects. The results showed that Ox-LDL suppressed the expression of SIRT1 in HUVECs in a dose- and time-dependent manner, and RSV restored the expression of SIRT1. After SIRT1 knockdown, the effect of RSV on the expression of LC3-II and p62 was abolished. The protective effects of RSV, which included upregulating the expression of mature cathepsin D and increasing cathepsin D activity, were also blocked and accompanied by accumulation of Ox-LDL in HUVECs after knockdown of SIRT1. Our results therefore demonstrated that SIRT1 is essential for the amelioration of Ox-LDL-induced lysosomal dysfunction and the impaired autophagic flux in HUVECs by RSV.

Although it has been reported that RSV protects HUVECs from Ox-LDL-induced oxidative damage by autophagy upregulation [13], the effects of Ox-LDL on autophagy in HUVECs remain elusive, this study for the first time demonstrated that Ox-LDL decreased autophagic flux in HUVECs in a concentration- and time-dependent pathway, and Ox-LDL-induced impaired autophagic flux leads to the accumulation of Ox-LDL in HUVECs. In this study, we found that the mechanism of Ox-LDL inhibited autophagic flux was due to the Ox-LDL-induced SIRT1-dependent lysosomal dysfunction. We also for the first time demonstrated the mechanism of RSV in protecting Ox-LDL-induced autophagic flux barrier in HUVECs with regulation of lysosomal function. RSV-induced Dil-Ox-LDL degradation in HUVECs disappeared when lysosomal degradation was blocked with Baf-A1, suggesting that lysosomal function is necessary for RSV-induced enhancement of autophagic flux in HUVECs.

In summary, in our present study, we demonstrated that Ox-LDL suppressed the expression of SIRT1, resulting in lysosomal dysfunction and impaired autophagic flux, which consequently led to the accumulation of Ox-LDL in HUVECs. RSV upregulated SIRT1 expression and restored the biological function of lysosomes, enhanced the autophagic flux, and increased Ox-LDL degradation through the autophagy-lysosomal pathway. This study found novel insights into the role of disordered autophagic flux in development of atherosclerosis. Our data also indicated that manipulation of lysosomal function might be a promising therapeutic target for the treatment of atherosclerosis, and RSV might be a promising drug in treatment of atherosclerosis with upregulation of autophagic flux in HUVECs through improvement of lysosomal function.

## Abbreviations

RSV: Resveratrol
HUVECs: Human umbilical vein endothelial cells
SIRT1: Sirtuin 1
p62: Sequestosome 1
BafA1: Bafilomycin A1.
